# Uptake of Hydrogen Peroxide from the Gas Phase to Grain Boundaries: A Source in Snow and Ice

**DOI:** 10.1021/acs.est.3c01457

**Published:** 2023-07-27

**Authors:** Angela C. Hong, Thomas Ulrich, Erik S. Thomson, Jürg Trachsel, Fabienne Riche, Jennifer G. Murphy, D. James Donaldson, Martin Schneebeli, Markus Ammann, Thorsten Bartels-Rausch

**Affiliations:** † Department of Chemistry, University of Toronto, Toronto, Ontario M5S 3H6, Canada; ‡ Laboratory of Atmospheric Chemistry, Paul Scherrer Institute, Villigen PSI CH-5232, Switzerland; § Department of Chemistry and Molecular Biology, Atmospheric Science, University of Gothenburg, Gothenburg SE-41296, Sweden; ∥ WSL Institute for Snow and Avalanche Research SLF, Davos Dorf CH-7260, Switzerland; ⊥ Department of Physical and Environmental Sciences, University of Toronto Scarborough, Toronto, Ontario M1C 1A4, Canada

**Keywords:** Arctic, snow, oxidation, chromophore, H_2_O_2_, HOOH, atmospheric oxidant

## Abstract

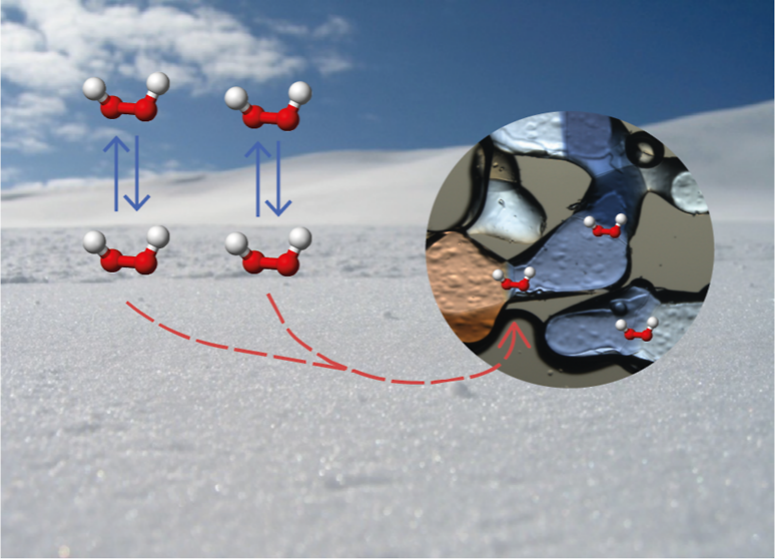

Hydrogen peroxide is a primary atmospheric oxidant significant in terminating gas-phase chemistry and sulfate formation in the condensed phase. Laboratory experiments have shown an unexpected oxidation acceleration by hydrogen peroxide in grain boundaries. While grain boundaries are frequent in natural snow and ice and are known to host impurities, it remains unclear how and to which extent hydrogen peroxide enters this reservoir. We present the first experimental evidence for the diffusive uptake of hydrogen peroxide into grain boundaries directly from the gas phase. We have machined a novel flow reactor system featuring a drilled ice flow tube that allows us to discern the effect of the ice grain boundary content on the uptake. Further, adsorption to the ice surface for temperatures from 235 to 258 K was quantified. Disentangling the contribution of these two uptake processes shows that the transfer of hydrogen peroxide from the atmosphere to snow at temperatures relevant to polar environments is considerably more pronounced than previously thought. Further, diffusive uptake to grain boundaries appears to be a novel mechanism for non-acidic trace gases to fill the highly reactive impurity reservoirs in snow’s grain boundaries.

## Abstract 2

This work shows that hydrogen peroxide readily enters grain boundaries in ice and snow directly from the atmosphere. Accelerated reaction rates in these reservoirs have been described with an impact on air quality and snow composition.

## Introduction

Hydrogen peroxide (H_2_O_2_) is a central inorganic oxidant in the hydrosphere and the atmosphere.^1^ In snow, it acts as a chromophore and a precursor for HO_*x*_.^2−5^ Temperature-dependent uptake and emission of H_2_O_2_ from snow alter gas-phase concentrations on daily timescales.^6,7^ Recent interest in the multiphase phase chemistry of H_2_O_2_ was sparked by a report on the autonomous formation of H_2_O_2_ at the interface of micron-sized droplets.^8,9^ In ice and snow, grain boundaries and their junctions provide micron-sized confined environments hosting impurities and multiphase reactions.^10−14^ In particular, oxidation reactions such as that of H_2_O_2_ with benzoic acid are highly accelerated in these locations in ice.^15^ We, therefore, investigate whether the uptake of H_2_O_2_ from the gas phase is a source of H_2_O_2_ in grain boundaries of snow. Its presence there might help better assess the environmental impact of the previous findings on accelerated reactivity.

An environmental source of chemical impurities in ice clouds, snowflakes, or snowpacks on the ground is the scavenging of trace gases.^14^ Adsorption at the surface routes in forming hydrogen bonds at the interface and is fundamentally identical for snow and ice. At environmental trace gas concentrations, it quickly leads to an equilibrium surface coverage of the adsorbate as a function of temperature and partial pressure.^16,17^ Previous work has characterized this adsorption equilibrium for H_2_O_2_ at temperatures up to 223 K with relevance as a loss process of this atmospheric oxidant to ice clouds in the upper troposphere.^18,19^ In this work, we quantify the adsorption of H_2_O_2_ to ice for higher temperatures that are most relevant for polar and alpine environments where snow is present, such as during Arctic spring, a period with significant active photochemistry in snow.

Adsorbates may also diffuse along grain boundaries of ice and snow.^14^ For acidic trace gases, such as HNO_3_,^20,21^ HCl,^21,22^ HONO,^23^ and SO_2_,^24^ this loss process was proposed to explain an uptake acting on longer time scales than adsorption,^25^ but direct evidence is missing. Interestingly, weaker organic acids, such as acetic and formic acid, adsorb to the air–ice interface without diffusing into grain boundaries.^26^ X-ray excited electron spectroscopy work revealed that strong acids such as HNO_3_ and HCl interact with the air–ice interface forming liquid-like regions, while formic acid and acetic acid do not.^27−31^ H_2_O_2_ was chosen because it has a high solubility in water, and this work provides clear experimental evidence of enhancing the overall uptake of H_2_O_2_ to snow by diffusion into the grain boundaries despite its low acidity.

To discern the effect of the ice microstructure on the uptake of H_2_O_2_, we have machined a novel flow reactor system featuring a drilled ice flow tube (DIFT). We use two distinct ice morphologies for the DIFT: monocrystalline (MC) ice, which is characterized by large crystals domains with a reduced grain boundary network, and polycrystalline (PC) ice, which has relatively more and smaller crystal domains and a more extensive grain boundary network within a comparable volume.

## Materials and Methods

The experiment consists of an H_2_O_2_ source to provide a steady gas-phase concentration of H_2_O_2_ in humidified N_2_ carrier gas, a flow tube where the H_2_O_2_ is passed over an ice sample that can be by-passed, and an H_2_O_2_ detector to monitor changes to the gas-phase H_2_O_2_ concentration in the carrier gas downstream of the flow tube. The flow tube was a DIFT, where a hole is bored through an ice block or an ice-coated-wall flow tube. A schematic of the experimental setup is shown in the Supporting Information (Figure S1).

### Preparing Drilled Ice Flow Tubes

The ice block characterized by large crystal domains (MC) has been purchased from the ICE Factory of Switzerland (www.icefactory.ch). The purchased ice block is not treated before use. Polycrystalline ice (PC) is grown by freezing ultraclean water (Millipore Milli-Q, 0.05 μS) at 253 K. From each MC and PC sample, one drilled ice flow tube (DIFT) is made by cutting the respective ice into blocks of 10 cm lengths and drilling a 6 mm borehole longitudinally through the middle of each block. At each end, the boreholes were widened to approximately 9 mm to attach fittings by screwing in pre-heated threads (Bola, Germany), which temporarily melts the ice and, upon refreezing, creates gas-tight connections. The connections also leave the inner borehole diameter flush with the inner fitting diameter to allow for smooth flow transitions. Additional water is frozen around the fitting external to the connector to increase durability. The DIFT boreholes are 80 mm long. Four experiments were done with one DIFT tube each on 2 days. Experiments started with the lowest H_2_O_2_ concentration, which then increased from experiment to experiment. DIFT tubes were sealed and stored at −20 °C overnight. Before the first experiment each day, the tubes were purged with humidified carrier gas for 30 min. Between experiments on the same day, desorption experiments served to purge the tubes (see Results and Discussion).

### Imaging Drilled Ice Flow Tubes

Thin DIFT cross-sections (250 μm) are prepared after drilling and imaged using a compound optical microscope with a nadir polarized light source as described earlier.^32,33^

### Coated Wall Flow Tube

Coated wall flow tube experiments are done in quartz glass tubes with 8 mm inner diameters. The inner surface of the quartz tube is covered with ice.^34^ Briefly, the quartz tube is internally etched with a solution of 5% hydrofluoric acid in water and subsequently is rinsed several times with purified water (Millipore Milli-Q, 0.05 μS). After rinsing, 8–10 mL of ultra-pure water (for high-performance liquid chromatography use, Fluka 14263) or laboratory Milli-Q water (Millipore Milli-Q, 0.05 μS) are pipetted into the quartz tube. Excess water is removed by orienting the quartz tube vertically for 1 min and draining. An ice film is formed at 258 K by slowly rotating the coated flow tube in a snuggly fitted double-mantled cooling jacket. This procedure results in thin ice films with an average thickness of 10 μm, as determined by mass. Quartz tubes of either 80 or 45 cm in length are used in this work. Data obtained with ice made from ultra-pure and Milli-Q water are in excellent agreement. For each experiment, a new coating was prepared.

### H_2_O_2_ Source

For the DIFT experiments, H_2_O_2_ is introduced to the experiment by adding a small stream of N_2_ carrier gas over a frozen solution of 30% H_2_O_2_ (Perhydrol p.a., 7209 Merck) at 248 K. For coated wall flow tube experiments, gas-phase H_2_O_2_ is dosed from a custom-built permeation tube. For this, a liquid solution of 30% H_2_O_2_ (Perhydrol p.a., 7209 Merck) fills a 17.4 cm long, thin-walled fluorinated ethylene propylene (FEP) tube sealed at both ends. The permeation tube is placed in a commercial permeation oven (VICI Dynacalibrator) at 323 K. A flow of pure nitrogen (Carbagas 99.9995% purity) through the permeation oven transports the gas phase H_2_O_2_ into the experimental setup.

### Detection of H_2_O_2_

Gas-phase H_2_O_2_ is measured using a commercial H_2_O_2_ analyzer with a detection limit of 100 ppt, corresponding to 2.5 × 10^9^ molecules per cm^3^ at 298 K and 1 atm (Aero-Laser, Germany, AL2021). The instrument relies on a fluorometric method, by which H_2_O_2_ is stripped from the gas phase in a glass coil and subsequently reacts with *p*-hydroxyphenyl acetic acid and peroxidase forming a fluorescence dimer. This method detects all peroxides in the solution. The reagents used for the analyzer were: Potassium phthalate (monobasic, 96148 Fluka), EDTA (Titriplex III p.a., 1.08418 Merck), NaOH (1 N, 71463 Fluka), formaldehyde (37 wt % in water, 252549 Sigma-Aldrich), *p*-hydroxyphenyl acetic acid (98%, H5,000-4 Sigma-Aldrich), and peroxidase (from horseradish, P8250 Sigma-Aldrich). The reagents are dissolved in double distilled water (3478.2 Roth). The instrument is calibrated with diluted liquid solutions of H_2_O_2_ (Perhydrol 30% p.a., 7209 Merck), which are titrated against a permanganate solution (permanganate 1/500 mol 38136 Fixanal) of certified concentration.

### Flow System

The tubing of the flow system consisted of perfluoro-alkoxy copolymer (PFA). The flow through the coated wall flow tube is controlled by the H_2_O_2_ analyzer and set to either 2000 mL min^–1^ STP (standard temperature and pressure: 273.15 K and 1 bar) or 500 mL min^–1^ STP, respectively. The flow through the permeation source is constant at 1500 mL min^–1^ STP. For the drilled flow tube experiments, the flow via the H_2_O_2_ source is set to 20 mL min^–1^ and mixed with a humidified flow of 215–230 mL min^–1^ N_2_. After passing the drilled flow tube or by-pass, the gas flow was diluted with 1900–2000 mL min^–1^ to quickly transport the H_2_O_2_ to the analyzer operated with a flow of 1500 mL min^–1^.

## Results and Discussion

### Characterizing Grain Boundaries

**Figure 1:**
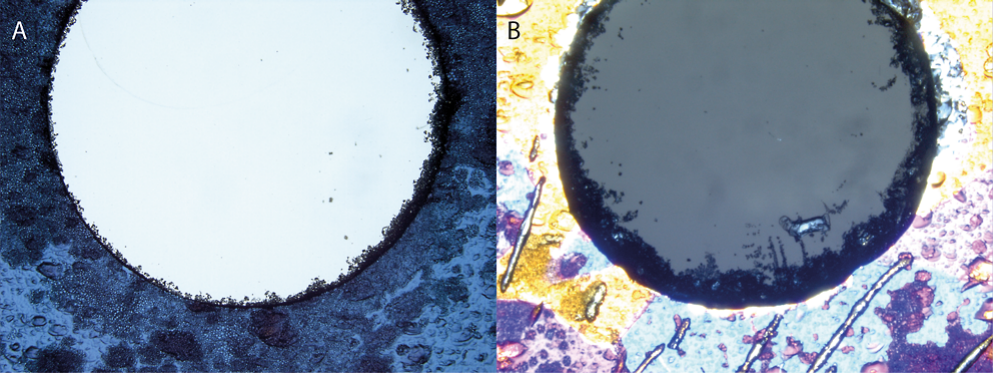
Thin cross-sections as obtained by polarization microscopy of MC ice (A) and the increased polycrystallinity of PC ice (B). The imaged boreholes have a 6 mm inner diameter. Individual crystals appear as uniformly colored grains. The images further show granular, dark-shaded features in large fractions of the image that appear to originate from debris associated with the microtome-cutting procedure. These were not visually detectable following the uptake-desorption experiments.

Figure 1 shows microscopy images of MC- and PC-DIFT thin cross-sections imaged using cross-polarized light. Due to the birefringent nature of ice, different crystal orientations are visualized as distinct color regions when viewed with polarized light.^33,35^ The PC-DIFT sample (Figure 1B) shows nine distinct grain areas of different colors. The MC-DIFT image (Figure 1A) displays much more homogeneity, i.e., a single ice crystal surrounding the borehole in this section. The figure confirms that the PC-DIFT sample contains relatively more single crystals and, thus, a more extensive grain boundary network reaching the air–ice interface than the MC-DIFT sample for the same cross-sectional area. The image in Figure 1B shows 6 grain boundaries at the flow tube’s air–ice interface of 6 mm diameter (or 19 mm length). The corresponding 0.3 grain boundaries per mm lie within the range for natural snow. Synchrotron-based diffraction-contrast tomography images indicate 0.5–2 grain boundaries per mm for hardened, aged snow.^36,37^ Freshly fallen surface snow is less dense and can be expected to have fewer grain boundaries, but to our knowledge, data are unavailable. In the following, we describe the fate of H_2_O_2_ upon adsorption to these ice samples with different grain-boundary densities.

### Uptake to Grain Boundaries and the Surface

**Figure 2:**
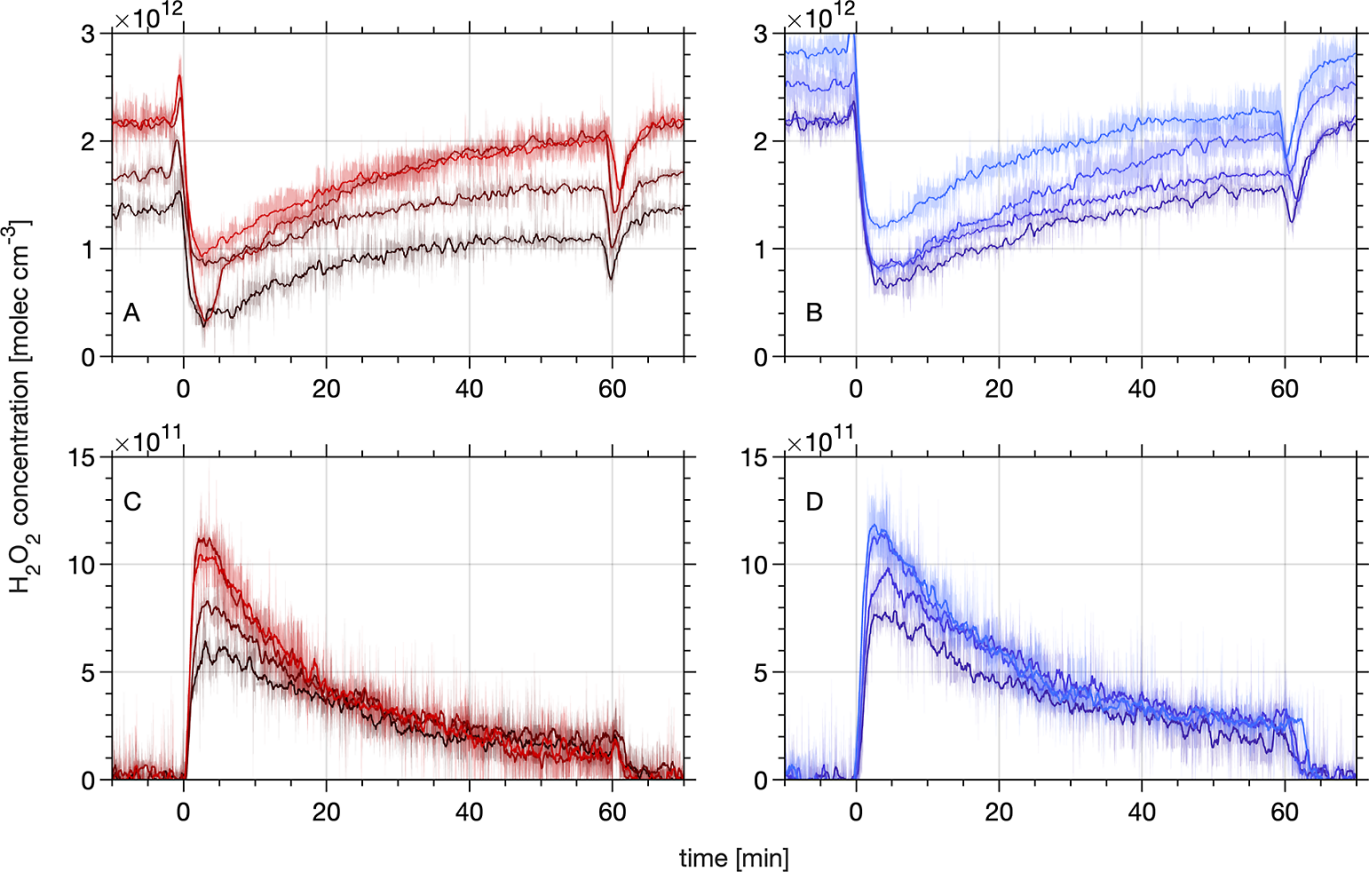
H_2_O_2_ uptake to and release from MC-DIFT (solid red lines) and PC-DIFT (solid blue lines) as a function of time at 253 K. Before *t* = 0 min and after *t* = 60 min, the gas flow is directed through the bypass. Panels (A,B) show the loss of H_2_O_2_ to the ice from the carrier gas during uptake experiments. The short dip in signal intensity at *t* = 60 min is attributed to H_2_O_2_ adsorbing at the walls of the dosing line. Panels (C,D) show the time traces of subsequent H_2_O_2_ desorption from the ice to the carrier gas. Colors denote the 4 repetitions for each MC- and PC-DIFT, respectively. The color density indicates the order of the repetitions at varying concentrations, with the darkest lines showing the first run. Each line shows the average of two measurements, and the shaded areas represent the standard error and, thus, indicate the uncertainty of data processing.

The upper panels in Figure 2 display uptake curves, that is, the change in the gas-phase H_2_O_2_ number density in the carrier gas evolving upon contact with the ice for the MC-DIFT (Figure 2A) and PC-DIFT (Figure 2B) experiments. The ice is exposed to between 1.4 × 10^12^ and 2.8 × 10^12^ molecules H_2_O_2_ cm^–3^ in the carrier gas. The flow tubes were bypassed at *t* < 0 min, and the humidified carrier gas with H_2_O_2_ was fed directly to the analyzer. At *t* = 0 min, the gas flow was redirected through the flow tubes, and the uptake curves reveal a fast loss of gas phase H_2_O_2_. The uptake curves slowly recover towards the initial gas-phase H_2_O_2_ concentration until the gas flow is directed again via the bypass at *t* = 60 min. Focusing on the recovery level reached toward the end of the experiment’s time scale reveals that the ongoing net uptake of H_2_O_2_ in the PC-DIFT is clearly more pronounced than in the MC-DIFT. This difference in the uptake behavior of H_2_O_2_ for the PC-DIFT compared to the MC-DIFT is attributed to a more extensive network of grain boundaries at the air–ice interface (Figure 1). The diffusion into such reservoirs has been proposed as a candidate to explain the slow, long-term recovery of uptake curves.^25^ This work links the direct observation of grain boundary content in ice samples with the diffusive loss. We want to stress the environmental relevance of this direct experimental evidence for H_2_O_2_ uptake from the gas phase to the grain boundaries, where H_2_O_2_ is a potent oxidant.^15^ It is important to note that the relevance of diffusive uptake varies not only with grain boundary content but also from species to species. Bartels-Rausch et al.^26^ showed that for the non-acidic volatile organics acetone and methanol, diffusion into grain boundaries is not a vital loss process in snow. The direct and long-term uptake of H_2_O_2_ from the gas phase is the first evidence of a non-acidic trace gas entering grain boundaries, casting questions on the importance of acidity as a driving factor.

### Reversibility of Uptake

We further explore the impact of grain boundaries on the reversibility of H_2_O_2_ uptake to MC- versus PC-DIFT. The lower panels in Figure 2 show desorption curves, where humidified carrier gas without added H_2_O_2_ was passed via the DIFTs that had previously been exposed to H_2_O_2_ (upper panel). The desorption curves probe the release of H_2_O_2_ from the ice samples. They show similar shapes with rapid increases of the gas-phase H_2_O_2_ concentration when the carrier gas flow through the DIFTs is initiated at *t* = 0 min. A slow decaying release follows for both the MC-DIFT and the PC-DIFT. The shape similarity between such uptake and desorption curves has previously been noted.^38^ Generally, the magnitude of H_2_O_2_ release observed during the desorption experiments scales with the gas-phase concentration of H_2_O_2_ that the DIFTs were exposed to during the uptake experiments. The highest release of H_2_O_2_ to the gas phase of 3.0 × 10^11^ molecule s^–1^ cm^–2^ (light red) and 3.3 × 10^11^ molecule s^–1^ cm^–2^ (light blue), yielded gas-phase maximum concentrations of 11 × 10^11^ molecule cm^–3^ (light red) and 12 × 10^11^ molecule cm^–3^ (light blue) in the desorption experiments for the MC- and PC-DIFT, respectively. These were observed in runs that followed uptake experiments with the highest gas-phase concentrations (light red and light blue in the upper panels of Figure 2). For both samples, the release of H_2_O_2_ is not complete after 60 min, indicating that the release occurs at a slower apparent rate than the uptake. The median recovery is 69% for experiments with MC-DIFT, significantly different from about 56% recovery determined for the PC-DIFT (see Supporting Information, Figure S3). A qualitative explanation for the longer residence time during the recovery experiments might be that in this mode, the concentration gradient drives diffusion back toward the air–ice interface, where the carrier gas passes the ice, and deeper into the ice. During the uptake experiments, the concentration gradients drive the net diffusion of H_2_O_2_ exclusively from the air–ice into the bulk ice interior. From the surface, H_2_O_2_ readily desorbs and is carried away by the gas stream once the desorption experiment begins. This leads to changes to the concentration gradients that drive bulk diffusion and a diffusive flux in the reverse direction toward the carrier-gas–ice interface establishes. However, because the H_2_O_2_ concentrations remain low in the interior of the DIFT tube bulk ice, diffusion away from the air–ice interface also continues. The partial irreversibility on the time scales of the uptake agrees with Conklin et al.,^38^ who found between 33 and 47% recovery. That their recovery is lower than that found in our PC-DIFT experiments might be due to a different grain boundary density in their samples and their longer experimental time scales that would allow for more extensive diffusion. Also, differences in pressure and flow tube geometry between individual flow tube studies, where the transport and retention of trace gases involve multiple re-adsorption steps, might hamper direct comparison. Nevertheless, Conklin et al.^38^ used sintered ice spheres that likely provide a significant but unknown grain boundary density.^33^ Interestingly, a different behavior was observed for the acidic HCl. McNeill et al.^39^ concluded that HCl is reversibly adsorbed to grain boundaries and observed full recovery within experimental time scales of up to 50 min at −60 °C.

### Quantifying the Diffusive-like Uptake

**Figure 3:**
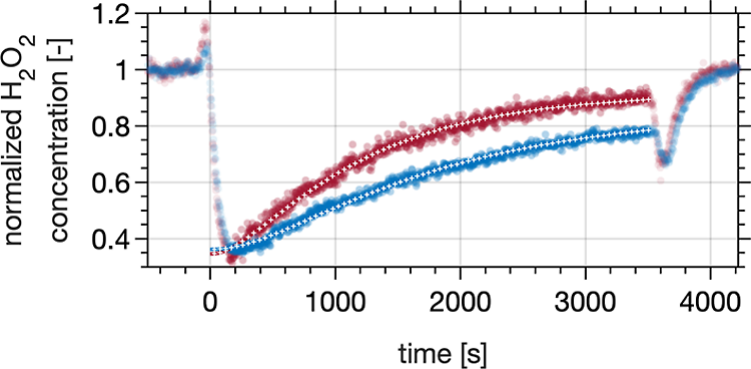
Uptake curves for MC-DIFT (red circles) and PC-DIFT (blue circles). The DIFTS were exposed to the H_2_O_2_ in the carrier gas at *t* = 0. The results from 4 individual experiments were normalized to the H_2_O_2_ gas-phase concentration before *t* = 0 and averaged. Filled circles denote the data that were fitted (white lines).

Our observation that grain boundaries in polycrystalline ice provide a sink for gas-phase H_2_O_2_ motivates quantification by comparing the observed uptake curves with theoretical predictions. Such comparisons have been used to identify the uptake mechanism at a wide range of experimental settings and time scales, tremendously impacting the shape of uptake curves.^25^ In Figure 3, we compare the experimental uptake curves to a kinetic model with coupled adsorption and diffusive uptake. Equation 1 gives the time-dependent H_2_O_2_ number density exiting the DIFT (*n*_DIFT_(*t*)) normalized to the initial concentration (*n*_0_) for the coupled adsorption-diffusion model as detailed in the Supporting Information (Supporting Information, “The resistor model”).^40−42^ Here, the length of the DIFT is denoted by *x*, the surface area of the DIFT borehole by *A*, the volume of DIFT borehole is *V*, ν̅ is the mean thermal velocity of the gas molecules, *u* is the linear gas velocity, and *t* the time. The mass accommodation coefficient α_c_ describes the fraction of molecules entering the condensed phase relative to those colliding with the interface. The time constant of Langmuir adsorption is denoted by λ. The free parameter we determine by fitting in this work is the product *H*√*D*, where *H* is the dimensionless Henry’s solubility law constant and *D* is the H_2_O_2_ diffusion constant in the solid phase. The solubility *H* is defined as the ratio between condensed-phase concentration and gas-phase concentration of a species.^43^
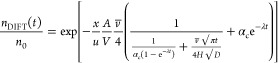
1

Figure 3 presents the asymptotic fit to the last 500 s of the uptake curves and shows the excellent capture of the observations by the coupled kinetic model. The agreement confirms that the H_2_O_2_ air–ice interaction can be understood as a combination of adsorption to the surface and the diffusive loss from the surface acting on longer time scales. We derive 3 and 7 cm s^–1/2^ for *H*√*D* for the MC- and PC-DIFT uptake curves from the fitting. That the value of *H*√*D* is larger for the PC-DIFT compared to the MC-DIFT by a factor of about two clearly shows the importance of grain boundaries for long-term, diffusive uptake of H_2_O_2_ into the ice matrix. In this analysis, the derived *H*√*D* must be understood as a combination of a grain-boundary-related *H*√*D* and the *H*√*D* in crystalline ice. Taken that the solubility and the diffusivity of H_2_O_2_ in the ice crystal and the grain boundary are identical for both DIFT tubes, we assign the difference in the *H*√*D* term to varying grain boundary numbers in both samples. This implies that both the loss rate of H_2_O_2_ and the apparent solubility are enhanced in environmental ice and snow as a function of grain boundary content. In the following, we estimate *H*√*D* values for the diffusive loss of H_2_O_2_ into single crystalline ice to support the hypothesis that this long-term loss process cannot explain the derived *H*√*D* alone. Neither the solubility nor the diffusivity of H_2_O_2_ in single crystalline ice is known. Supposing H_2_O_2_ has a similar solubility and diffusivity in ice as HCl, *H*√*D* of 0.2–2.8 cm s^–1/2^ can be calculated at −25 °C based on published solubility and diffusivity data.^44^ The scatter reflects experimental uncertainty. The similarity of the observed *H*√*D* in MC-DIFT with this estimate indicates that diffusion into the crystalline ice phase could explain a significant fraction of the long-term trend observed in MC-DIFT experiments but not that in PC-DIFT. However, the solubility of HCl in ice is exceptionally high compared to HNO_3_, another trace gas of atmospheric relevance. Solubilities in single crystalline ice (solid solution) of 0.1 mol/L for HCl versus 0.001 mol/L for HNO_3_ have been found at partial pressures of 0.2 × 10^–3^ and 0.7 × 10^–3^ Pa and temperatures of −25 °C.^44,45^ Just as H_2_O_2_, HNO_3_ is highly soluble in water^43^ and, therefore, we estimate the solubility of H_2_O_2_ in ice in the following based on HNO_3_ solid solution data. For HNO_3_, *H*√*D* values of 0.008–0.02 can be derived, which are significantly smaller than the results even for MC-DIFT. If we assume that the solubility of H_2_O_2_ in crystalline ice is closer to HNO_3_ than to HCl, the contribution of the solid-state diffusion to the long-term uptake in our experiments is negligible, and consequently, diffusion into grain boundaries would dominate the long-term uptake of H_2_O_2_ to ice in both samples.

The direct comparison of the uptake to samples that differ in grain-boundary density and that the diffusive loss is larger than expected for diffusion into the ice crystal strongly suggests that the observed long-term DIFT uptake is driven by diffusion into grain boundaries. The preference of H_2_O_2_ for grain boundaries in environmental snow and ice might result in significant variations in the local concentration of H_2_O_2_. This is relevant as such non-uniform chemical morphologies and highly concentrated patches of reactants at grain boundaries and elsewhere in the ice have recently been shown to impact chemical reactivity in frozen systems strongly.^13,15,46^ Grain boundaries in natural snowpacks occur at the contact face of individual snow grains.^47^ As grain size, shape, and arrangement change with time in snowpacks (metamorphism),^48^ detailed studies observing the impact of grain boundaries at different stages of metamorphism are timely to elucidate the mechanism and the environmental impact more extensively.

### Temperature Trend of Surface Adsorption

**Figure 4:**
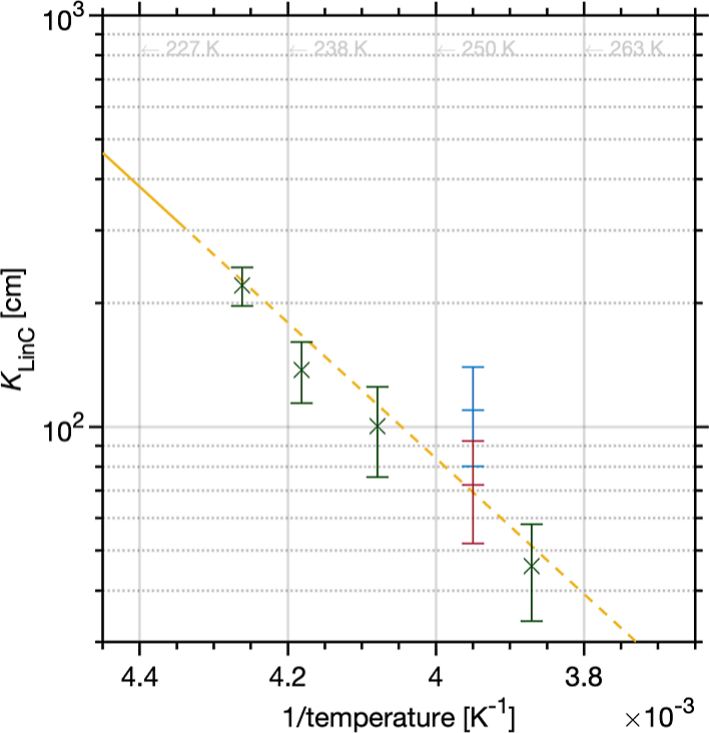
Linear Langmuir partitioning coefficient of H_2_O_2_ vs temperature, including data for the coated wall flow tube (green markers), MC-DIFT (red marker), and PC-DIFT (blue marker). Error bars represent one standard deviation from repeated experiments. Also shown is a parameterization of the partitioning coefficient based on published data^18^ (solid yellow line) and its extrapolation to warmer temperatures (dashed yellow line).

Given that the uptake curves are well described by a combination of long-term diffusion and short-term adsorption (eq 1, Figure 3), numerically integrating the initial part of the uptake curves further allows us to quantify the H_2_O_2_ partitioning equilibrium between the ice surface and the gas phase (Supporting Information, Figure S4). Figure 4 shows the temperature trend and the magnitude of the adsorption equilibrium at temperatures from 258 to 235 K derived in coated wall flow tube experiments and in the MC-DIFT and the PC-DIFT. The observed negative temperature trend of the partitioning coefficient is strong experimental proof for an adsorption process during this initial uptake regime that can be well described with a simple Langmuir model.^16,18^ The partitioning coefficient, *K*_LinC_, is defined as the surface concentration of the adsorbed H_2_O_2_ in molecules cm^–2^, divided by the gas-phase concentration in molecules cm^–3^. The ice–air interface has become more disordered in this temperature range.^14^ The consistent negative temperature trend with extrapolated results from an earlier low-pressure coated wall flow tube study between 203 and 233 K^18^ indicates that the presence of this disordered interface [also referred to as quasi-liquid-layer (QLL), pre-melting] does not change the uptake behavior of H_2_O_2_ up to 260 K.

The reported partitioning coefficients between 258 and 235 K make H_2_O_2_ one of the stickier atmospheric trace gases compared to volatile alcohols, aldehydes, and ketones. Its partitioning coefficient is 2–3 orders of magnitude larger than that of methanol or acetone with 10^0^ and 10^–1^ cm, respectively, at 240 K and even larger than that of acetic acid for which a partitioning coefficient of 10^1^ cm has been found at 240 K.^49^ For comparison, strong acids such as HCl have partitioning coefficients of 10^4^ cm at 240 K.^50^ The reported partitioning implies that a large fraction of H_2_O_2_ is found adsorbed to surface snow and not in the gas phase in this temperature range typical for the Arctic. A surface area of 59 cm^2^ per cm^3^ has been reported for freshly fallen snow,^51^ which yields 2 × 10^12^ molecules of H_2_O_2_ adsorbed per cm^3^ of snow at 260 K and 7 × 10^12^ molecules per cm^3^ at 240 K with the partitioning coefficients reported in this work; while the air above snow holds 10^9^ molecules cm^–3^. Because the air–ice interface of coated wall ice films is smooth on a molecular level,^16^ and that of the DIFTs can be expected to be so because any initial surface roughness and microstructures are generally smoothed by sintering with time,^52^ the adsorption process and the partitioning coefficients are invariant between ice and snow. Such gas-phase concentration of H_2_O_2_ used in this estimate is typical in the Arctic.^53^ For technical reasons, experiments have been done with higher H_2_O_2_ concentrations of 10^11^ molecules per cm^3^ in the coated wall flow tube experiments. Because surface coverages of H_2_O_2_ were low, in the 10^13^ molecules cm^–2^ range, extrapolation to the even lower environmental gas phase and surface concentrations is feasible.^17^ H_2_O_2_ and other polar trace gases adsorption saturate at a so-called monolayer coverage of ∼2 to 4 × 10^14^ molecules cm^–2^.^16,18^ In summary, the increase of H_2_O_2_ adsorbed to the snow by a factor of 3 when temperatures decrease by 20 K and the fast time response of the adsorbed fraction, as evident in Figure 1, is consistent with contributing to reported diurnal cycles of H_2_O_2_ in the polar regions.^6,7^ Jacobi et al.^7^ described the complex interplay of physical partitioning and snowpack chemistry in polar H_2_O_2_ observations. This study shows strong partitioning of H_2_O_2_ to the snowpack at high temperatures where it is subsequently H_2_O_2_ available for chemistry. The impact of surface adsorption can be expected to decrease with snow age as snow surface area reduces with time during metamorphism.^54^ The data in this work now give the basis to address the impact of various snow types and stages of metamorphism.

The uncertainty range of both DIFT data points overlap, indicating that H_2_O_2_ shows the same equilibrium adsorption behavior as the three types of ice used in this study. Uncertainty might come from the higher gas-phase and resulting surface concentrations of 1.3× 10^14^ to 2.6 × 10^14^ molecules cm^–2^ present during the DIFT experiments (see Supporting Information, “Results, data table”). At these surface coverages approaching a monolayer coverage,^18^ adsorbate–adsorbate interactions might start to alter the adsorption process.^17^ Additionally, diffusion into the sparse air pores (Figure 1) might contribute to the uncertainty. Nevertheless, this is direct experimental evidence that grain boundaries per se do not play an active role in the surface adsorption of H_2_O_2_ on short-time scales. Grains are considered large in coated wall ice films resulting in few grain boundaries,^39^ which cannot be visualized in these curved and thin samples. We, therefore, refrain from analyzing the diffusive uptake in more detail for these experiments. Comparison of the adsorptive surface coverages with the long-term diffusive uptake for the PC-DIFT (Figure 2B) emphasizes the importance of grain boundaries as a reservoir for the uptake of H_2_O_2_ from the gas phase in the environment. The diffusive loss term of 7 cm s^–1/2^ for *H*√*D* of the PC-DIFT corresponds to a flux of 28 × 10^12^ molecules per cm^3^ and per hour to the snowpack (See Supporting Information “Resistor Model”). For this snowpack, an H_2_O_2_ adsorption of 2 × 10^12^ molecules per cm^3^ of snow has been estimated above. The flux to the grain boundaries exceeds the number of surface adsorbed molecules by a factor of 10 each hour. Interestingly, in experiments with artificial snow Kerbrat et al.^23^ also found a 10 times increase compared to the surface adsorbed HONO each hour at 260 K. Apparently, the slower diffusive HONO loss of 1.6 cm s^–1/2^ for *H*√*D* reported by Kerbrat et al.^23^ for these snow samples and the lower surface partitioning^23^ result in the same relative increase. This back-of-the-envelope comparison illustrates the unmatched strong affinity of H_2_O_2_ for ice surfaces and the grain boundary network of snow. Similarly, a *H*√*D* of 0.02 cm s^–1/2^ for SO_2_, a weakly adsorbing trace gas,^19^ was found to explain the uptake to artificial snow with negligible surface adsorption. Kinetic modeling results revealed diffusive loss of HNO_3_ at 239 K and HCl at 213 K, corresponding to 10 times and 0.7 times the adsorptive surface coverage per hour, respectively.^21,39^ Detailed investigation of the relative importance of adsorption and grain boundary diffusion of these species is timely, with focus on the quantification of the grain boundary content in artificial and natural snow and wide variations of temperature and partial pressure. Our results put the relative importance of H_2_O_2_ diffusive loss on the level with the acidic trace gases, for which the observation of diffusive tailing in uptake curves has kicked off this line of research.^22^

## Supporting Information Available

Sketch of the experimental set-up, description of the data processing, box plot of the recovery results, derivation and results of the resistor model, and table with data (PDF)
