# Supplementary material for: Uptake of Hydrogen Peroxide from the Gas Phase to Grain Boundaries: A Source in Snow and Ice
Source: Environ Sci Technol. 2023 Jul 27;57(31):11626–33. doi: 10.1021/acs.est.3c01457 (PMC10413943; doi:10.1021/acs.est.3c01457)
Supplement: Supplementary file 1 — es3c01457_si_001.pdf [file es3c01457_si_001.pdf]

Supporting Information to

Uptake of hydrogen peroxide from the gas phase to grain boundaries: A source in snow and ice.

Angela C. Hong[1], Thomas Ulrich[2], Erik S. Thomson[3], Jürg Trachsel[4], Fabienne Riche[4], Jennifer G. Murphy[1], D. James Donaldson[1,5], Martin Schneebeli[4], Markus Ammann[2], and Thorsten Bartels-Rausch[2*]

[1] Department of Chemistry, University of Toronto, Toronto, ON M5S 3H6, Canada
[2] Laboratory of Atmospheric Chemistry, Paul Scherrer Institute, CH-5232 Villigen PSI, Switzerland.
[3] Department of Chemistry and Molecular Biology, Atmospheric Science, University of Gothenburg, SE-41296, Gothenburg, Sweden.
[4] WSL Institute for Snow and Avalanche Research SLF, CH-7260 Davos Dorf, Switzerland
[5] Department of Physical and Environmental Sciences, University of Toronto Scarborough, Toronto, ON M1C 1A4, Canada

*Corresponding author: Thorsten Bartels-Rausch (thorsten.bartels-rausch@psi.ch)

Summary

Number of pages: 9
Number of figures: 5
Number of tables: 2

Experimental, flow system

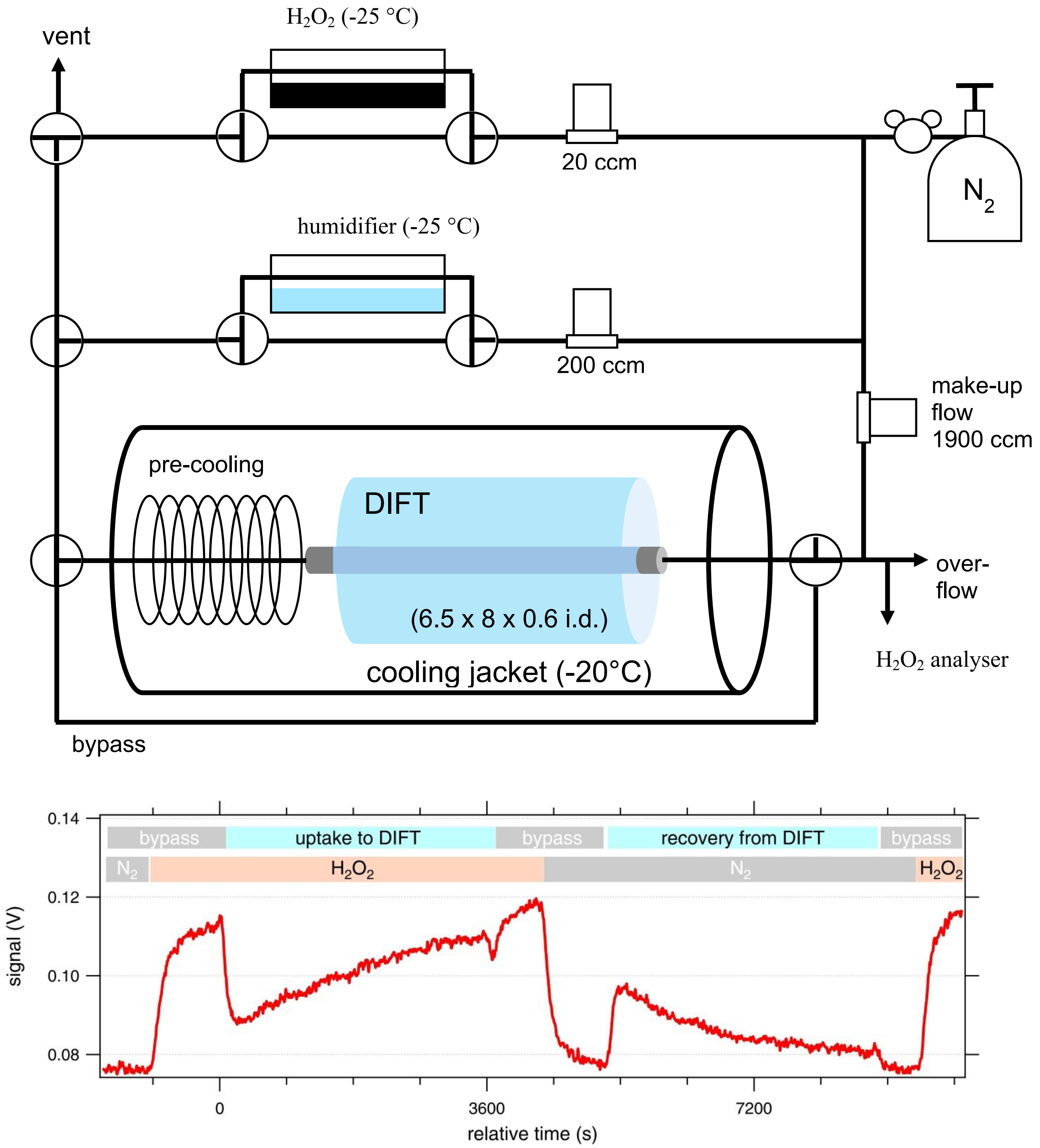

Figure SI-1. A schematic of the flow reactor system with the DIFT. All tubing consisted of PFA, and all gas flows were regulated by mass flow controllers (Brooks MFC or Kobold). Nitrogen (Carbagas 99.9995 % purity) was used as the carrier gas. The lower panel shows an experimental trace (in V corresponding to H_2O_2 number density) for a paired uptake-desorption experiment. Initially, N_2 gas flows through the bypass line to derive the instrumental background level. Then H_2O_2 is introduced, while the carrier gas continues to bypass the DIFT to derive the signal level corresponding to the concentration of H_2O_2 dosed to the carrier gas. For the uptake experiment, the flow of H_2O_2 in N_2 was directed from the bypass line into the DIFT at t=0 s. At the end of the uptake experiment, the flow is directed back to the bypass line. For the desorption experiment, N_2 gas is flowed first via the bypass line to flush the gas delivery lines. Then the carrier gas is passed via the DIFT. At the end of the desorption experiment, the flow is directed back to the bypass line.

Figure SI-1 shows the experimental setup. For an uptake experiment, the flow of H_2O_2 in humidified N_2 was diverted from the bypass line through a pre-cooled (253 K) PFA tubing housed within an ethanol-chilled cooling jacket and then entered the DIFT, which was placed within the same cooling jacket. The concentration of H_2O_2 exiting the flow tube was monitored over one hour, much longer than the approximately 2 s residence time of the carrier gas, using an online analyzer situated downstream of the DIFT. At the end of each uptake experiment, the flow is directed back to the bypass line. Subsequently, a desorption experiment is conducted by introducing pre-cooled and humidified N_2 gas into the bypass line and then into the DIFT while monitoring the peroxide signal. At the end of the desorption experiment, the flow is directed back to the bypass line. All experiments were done with one of the two DIFT tubes. Between experiments, the tubes were kept within the set-up with no gas flow, and at 253 K. Coated wall flow tube experiments were operated analogously.

Experimental, data processing

The direct observable in these experiments is changes to the gas-phase concentration of H_2O_2 exiting either the DIFT or the by-pass line. The uptake to the ice is quantified as the difference between the by-pass signal to the uptake signal and that of the desorption as the difference between the desorption signal and that of the baseline. The by-pass signal showed drifts upwards during the day due to conditioning. This trend in the concentration of H_2O_2 during the by-pass periods was accounted for by fitting and interpolating the by-pass periods directly before and after each uptake experiment (Fig. SI-2). There might further be small desorption of H_2O_2 from the transport wall lines after the experiments that only slowly ceases. A linear baseline fit when both the H_2O_2 source and the DIFT tube were bypassed accounted for this. A control experiment revealed 0.1×10^{12} molecules of H_2O_2 cm^{-3} released from the MC-DIFT into H_2O_2-free carrier gas. The PC-DIFT did not show such a release when H_2O_2-free carrier gas flowed through the DIFT. We assign this background to traces of peroxides being released from the MC-DIFT tube that might originate from earlier experiments performed with the same flow tube. This background was subtracted during data processing. We assign the higher scatter in the MC-DIFT recoveries to day-to-day variability in this background H_2O_2 release.

Results, drilled ice tubes

The total uptake of H_2O_2 to the ice for the 60 min experiments resulted in apparent surface coverages of 3×10^{14} molecules cm^{-2} to 6×10^{14} molecules cm^{-2} for the MC-DIFT and 8×10^{14} molecules cm^{-2} to 9×10^{14} molecules

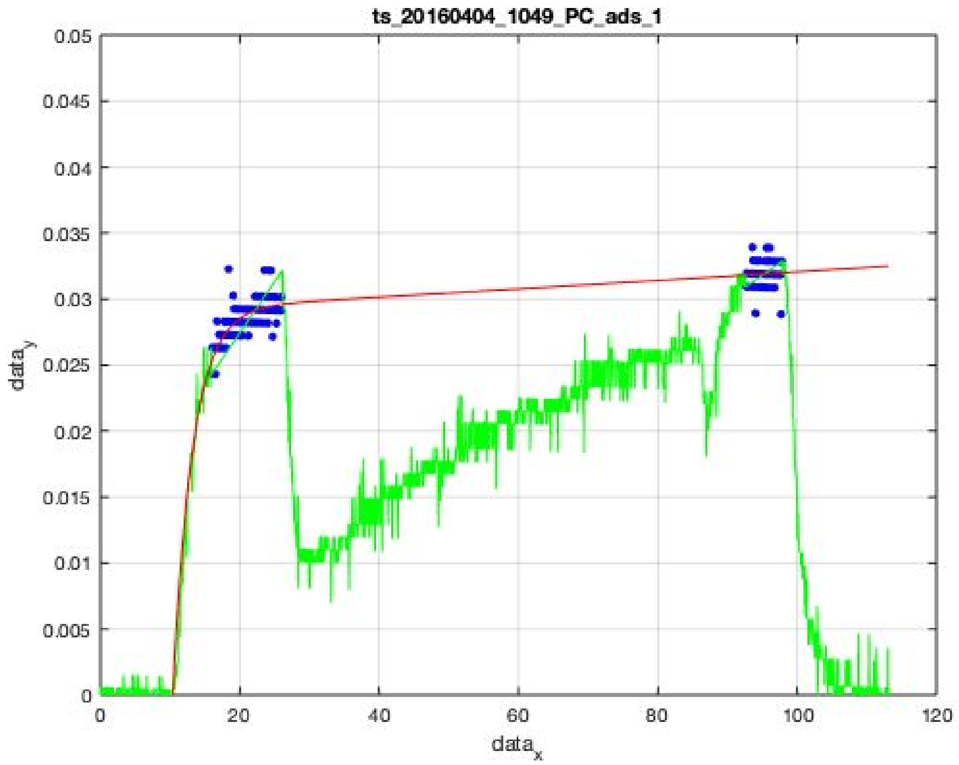

Figure SI-2: Typical H_2O_2 trace during an uptake experiment. The red line denotes the fit of the by-pass signal. Integration was done between this line and the H_2O_2 trace (green line) during 60 minutes.

cm^{-2} for the PC-DIFT. For this analysis, the area between the total uptake curves and an interpolation of the bypass signal was integrated between t= 0 minutes and t= 60 minutes and divided by the geometric surface area

of the DIFT. The apparent surface coverage quantifies the uptake without differentiating between adsorption to the surface and uptake to other reservoirs, formally expressing the total uptake as adsorption. This approach has no physical meaning. It serves to compare the observed uptake with results from other flow tube studies that lack a detailed analysis of the uptake mechanism and often report such apparent surface coverages.

The four repetitions of the experiment in the same MC- or PC-DIFT, respectively, are color-coded in Figure 2 of the manuscript. Of these four experiments in the MC- and PC-DIFT each, two had the same concentration of H_2O_2 in the gas phase, and the concentration was slightly varied for the other runs (see table SI-T2). The curves differ somewhat in their shape during the first 10 minutes of each experiment. We judge the reproducibility towards the end of each experiment at 60 min., the period from which we discuss the recovery level, as excellent. Uncertainty in these results comes mainly from instrumental variability and data processing. To assess the uncertainty from data processing, including the fits to account for shifts in the baseline and by-pass signal with time, each line represents the average of two data recordings and independent processing of the same experiment using both available channels of the H_2O_2 analyzer. The shaded area illustrates the deviations between these two data recordings, which we judge as minor.

Results, reproducibility

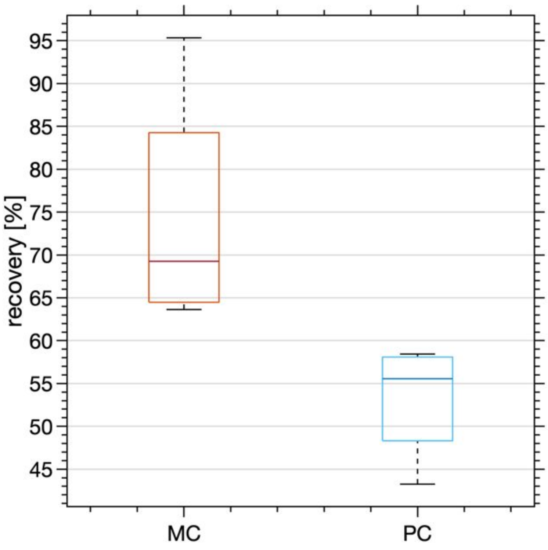

Figure SI-3. Box plots of the recovery for 4 MC- and 4 PC-DIFT experiments. Recovery is the ratio of H_2O_2 molecules emitted to the gas phase during the desorption experiments relative to that lost from the gas phase to the ice phase during the uptake experiments. The line in each box gives the median, the upper and lower edge of the box shows the 25th and 75th percentile, and the whiskers extend to the extreme data points.

Figure SI-3 shows the ratio of H_2O_2 molecules recovered during the desorption experiments relative to those lost during the adsorption experiments. Numerical integration over 60 minutes was performed on the paired uptake and desorption curves (see table SI-T2). The box plot of the four individual MC- and PC-DIFT experiments reveal that the median recovery is 69 % for experiments with MC-DIFT and significantly different from about 56 % recovery determined for the PC-DIFT. For each box in Fig. SI-3, the bottom and top edges indicate the 25th and 75th percentiles, respectively, and the whiskers extend to the extreme data points. The MC-DIFT recovery shows a larger scatter as compared to the PC-DIFT.

Results, coated wall flow tubes

Figure SI-4 shows the evolution of gas-phase H_2O_2 with time as derived in two typical experiments downstream of coated wall flow tubes. A linear fit through the long-term signal of the data is used, as illustrated for the coated wall flow tube experiments in Fig SI-4; the area between the uptake curve and this fit gives the number of molecules adsorbed. This approach is similar to those previously used by [1, 2]. This analysis yields surface coverages of 4×10^{13} molecules cm^{-2} at 235 K to 3×10^{12} molecules cm^{-2} at 258 K for these experiments.

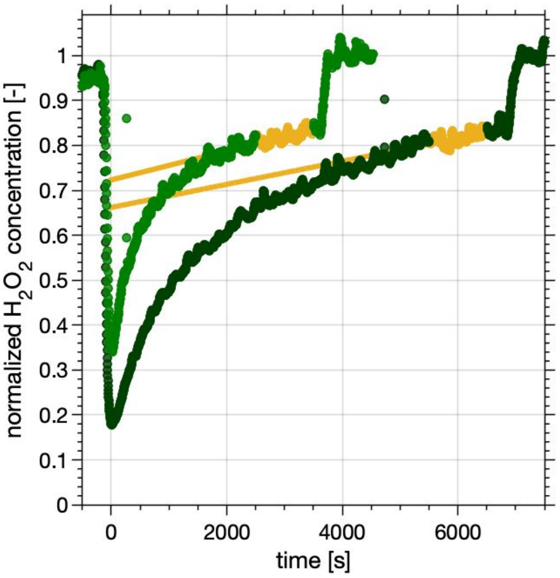

Figure SI-4: The gas-phase concentration of H_2O_2 downstream of a coated wall flow tube with time at 239 K (dark green dots) and 258 K (light green dots). Data are normalized to the H_2O_2 gas-phase concentration in the carrier gas. At time 0, the humidified carrier gas containing H_2O_2 was passed via the ice-coated flow tube and at 3500 s and 7000 s, respectively, again via the by-pass. The yellow marks indicate the data region that was linearly fitted. The area between the linear fit (yellow line) and the uptake curves denotes the initial, adsorptive uptake in analogy to the 2/3 procedure used by others [1, 2]. The ice-coated flow tube with a length of 80 cm was operated at atmospheric pressure and a carrier gas flow of 2 l min^{-1} STP. H_2O_2 concentrations were 0.4×10^{11} molecule cm^{-3} and 4.2×10^{11} molecule cm^{-3}, respectively

Reynolds numbers were calculated for both flow regimes, resulting in $Re = 440$ for 2000 ml min^{-1} STP and $Re = 110$ for 500 ml min^{-1} STP, indicating laminar flow throughout all experiments. The dew point of the gas flow was compared to the dew point of the ice with a commercial dew point sensor (EdgeTech Dewmaster) before each experiment. For this, the dew point of the gas flow was measured; subsequently, the dew point after the CWFT was measured and compared to the temperature inside the CWFT and the dew point of the gas flow. If necessary, the temperature of the CWFT was adjusted to the dew point of the water vapor in the gas flow. The temperature inside the coated wall flow tube was measured at experimental conditions with a type K thermocouple.

The resistor model

The detailed derivation of the kinetic model can be found in [3]. Briefly, we assume that the quasi-stationary condition is satisfied and that there is a uniform velocity profile through the flow tube (plug flow assumption), leading to a linear flow rate (u). Under this assumption, one expects an initial rapid drop in the trace gas number density due to adsorption. A first-order decay of the uptake towards zero follows this initial phase as the adsorption sites become filled. In Eq. 1, we model the H_2O_2 number density at the time (t) observed in the DIFT ($n_{DIFT}(t)$), normalized to its initial concentration (n_0). The uptake coefficient ($\gamma(t)$) describes the net probability that a molecule from the gas phase will enter the condensed phase as it collides with the surface. Note that, saturation effects and desorption from the surface result in a pronounced time dependency for the uptake coefficient [3].

$$\frac{n_{DIFT}(t)}{n_0} = exp\left(-\frac{xA\bar{v}}{uV4}\gamma(t)\right) \quad (1)$$

In addition to $\gamma(t)$, the residence time of H_2O_2 in the DIFT is also governed by known experimental variables, including the length of DIFT (x), the surface area of the DIFT borehole available for adsorption (A), the volume of DIFT borehole (V), and the mean thermal velocity of the gas molecules ($\bar{v}$). We assume that the net uptake coefficient is a composite of two independent loss pathways to the surface through Langmuir adsorption, given in Equation 3, and loss to the bulk via diffusion, given in Equation 4:

$$\gamma(\mathrm{t}) = \gamma_{surf}(t) + \gamma_{bulk}(t) \quad (2)$$

$$\gamma_{surf}(\mathrm{t}) = \alpha_c e^{-\lambda t} \quad (3)$$

$$\gamma_{bulk}(\mathrm{t}) = \frac{1}{\frac{1}{\alpha_c(1-e^{-\lambda t})} + \frac{\bar{v}}{4H}\sqrt{\frac{\pi t}{D}}} \quad (4)$$

To model the time-dependent surface uptake coefficient for H_2O_2 (γ_{surf} (t)), we assume a Langmuir adsorption isotherm, which describes gas uptake to a finite number of independently available, non-interacting surface sites (n_{sites}). Then $\gamma_{surf}(t)$ is given by the effective mass accommodation coefficient (α_c), modulated by a first-order decay of the number of available sites due to the previous adsorption. The mass accommodation coefficient describes the fraction of molecules entering the condensed phase relative to those colliding with interface from the gas phase. It relates to the uptake coefficient by $\gamma\ (t\rightarrow 0) = \alpha_c$. At the time limit t→∞, γ_{surf}=0 indicating the surface is at equilibrium, and there is no net surface uptake. This time dependency of γ_{surf} is captured in the time constant (λ) for Langmuir adsorption, which describes the characteristic time to reach surface equilibrium. The bulk uptake coefficient ($\gamma_{bulk}\ (t)$) that in principle captures diffusive loss into any bulk reservoir such as the ice crystal as well as grain boundaries is formulated using the "resistor" approach [4-6]. Diffusive loss from the gas phase is related to the mean thermal velocity of the gas ($\bar{v}$), dimensionless Henry's law coefficient parameterizing the solubility of H_2O_2 in bulk ice, and diffusivity (D) of H_2O_2 in bulk ice [3]. Since the surface accommodation and diffusion processes are contingent, their resistances are added in series. Substituting Equation (3) and (4) into (1), gives the overall uptake coefficient for our DIFT system, Equation 5. Similar equations describing uptake processes that couple the surface and bulk reservoirs have been derived elsewhere [6-9].

$$\gamma_{(t)} = \frac{1}{\frac{1}{\alpha_c(1-e^{-\lambda t})} + \frac{\bar{v}}{4H}\sqrt{\frac{\pi t}{D}}} + \alpha_c e^{-\lambda t} \quad (5)$$

To constrain the fits to Eq.5, we first fit the last 500 s of the uptake curves to the diffusive limit for the flow tube equation, where in the limit t → ∞,

$$\frac{n_{DIFT}(t)}{n_0} = exp\left(-\frac{xAH\sqrt{D}}{uV\sqrt{\pi t}}\right). \quad (7)$$

The parameters derived from these fits are presented in Table 1. A coupled adsorption-diffusion flow tube expression is used to fit the uptake curves over the entire period of observation (250 – 3500 s):

$$\frac{n_{DIFT}(t)}{n_0} = exp\left[-\frac{xA\bar{v}}{uV4}\left(\frac{1}{\frac{1}{\alpha_c(1-e^{-\lambda t})} + \frac{\bar{v}}{4H}\sqrt{\frac{\pi t}{D}}} + \alpha_c e^{-\lambda t}\right)\right] \quad (8)$$

The Hsqrt(D) was constrained to the 95% confidence interval derived from fits using Eq. 7. The resulting extracted fit parameters are displayed in Table 1. Interestingly, the predicted effective mass accommodation coefficient and lambda are similar for the MC- and PC-DIFT. This indicates that adsorptive processes are identical in adsorption probability and energetics, irrespective of the number of grain boundaries in the ice sample. Compared to the reported value of the mass accommodation coefficient of 0.2 for aqueous surfaces at 273 K [10], the results here reflect the strong influence of gas phase diffusion on the interface and also the fact that the gas undergoes multiple collisions with the flow tube in these experiments at atmospheric pressure.

Figure SI-5 presents an asymptotic fit to the last 500 s of the uptake curves. The equations and parameters derived from these fits are presented in Table 1. As expected, the parameterization for the solid-state diffusive limit (Eq. 7) fails to reproduce the uptake curves over the entire experiment for either the MC- or PC-DIFT results because the adsorptive uptake that dominates in the initial period of the uptake is neglected. The fits allowed deriving Hsqrt(D) that dominates the uptake at longer time scales for both types of DIFT tubes. The 95% confidence interval derived from the asymptotic fits was then used to constrain fits to the entire uptake curves (250 – 3500 s) using a coupled adsorption-diffusion flow tube expression (Eq. 8) in Fig. SI-5b and as discussed in the manuscript.

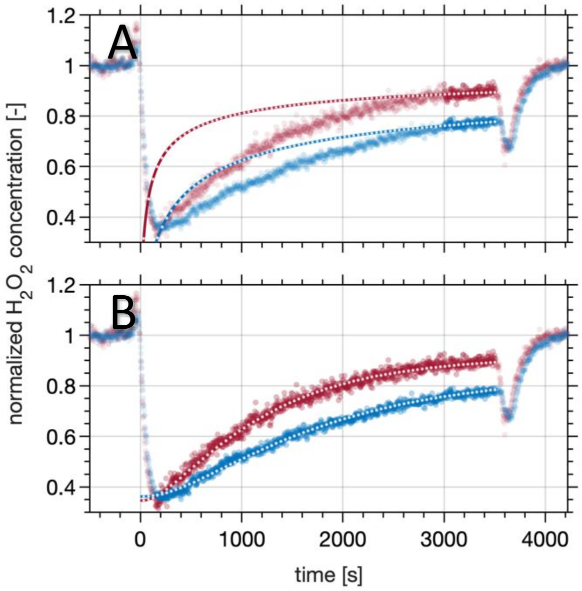

Figure SI-5. Uptake curves for MC-DIFT (red circles) and PC-DIFT (blue circles). The DIFTS were exposed to the H2O2 in the carrier gas at t = 0. The results from 4 individual experiments were normalized to the H2O2 gas-phase concentration before t = 0 and averaged. Shown in (A) are fits to Eq. 7 to retrieve Hsqrt(D). In (B), the fit to Eq. 8 is displayed, from which the effective mass accommodation coefficient is retrieved. Filled circles denote the data used to calculate the fitted (dotted) lines.

Table SI-T1. Estimated coefficients for parameterized flow tube equation modeling H_2O_2 uptake to the MC- and PC-DIFT* as driven by diffusion only (Equation 7), coupled adsorption-diffusion kinetics (Equation 8). Statistical significance is reported at the 95% confidence interval.

	MC-DIFT		PC-DIFT	
	Diffusion	Coupled	Diffusion	Coupled
α_c	-	3×10^{-5} $(2.92 – 2.98) \times 10^{-5}$	-	3×10^{-5} $(2.81 – 2.84) \times 10^{-5}$
λ (s^{-1})	-	1×10^{-3} $(1.23 – 1.26) \times 10^{-3}$	-	1×10^{-3} $(0.89 – 0.94) \times 10^{-3}$
H√D ($cm\ s^{-1/2}$)	3 (3.17-3.31)	3	7 (7.24 – 7.34)	7

* input based on experimental conditions
x = 8 cm
u = 14.7 cm s^{-1}
A = 15.1 cm^2
V = 2.26 cm^3
$\bar{v}$ = 3.97x 10^4 cm s^{-1}

Following [11] and [12], the kinetic H√D can be related to the partitioning coefficient to compare the adsorption and uptake at any time:

$$K_{total}(t) = K_{LinC} + 2 \times H \times \sqrt{\frac{D \times t}{\pi}}$$

Results, data table

Table SI-T2 summarizes the findings that are the basis for Figure SI-3 and Figure 4. Individual results for the DIFT experiments are listed, and averages of these values are given in Figure 4. For the coated wall flow tube experiments, the averages of the KLinC as shown in Figure 4 are listed. The number in brackets gives the number of repetitions. For the coated wall flow tube experiments, the concentration ranges of the experiments are also given.

Uptake experiment	Gas concentration molecules cm^{-3}	Surface concentration molecules cm^{-2}	Recovery %	KLinC cm
**Coated wall (2), 235 K**	0.7 - 1.0 x 10^{11}	1.6 - 2.1 x 10^{13}	-	220
**Coated wall (7), 239 K**	0.2 - 4.3 x 10^{11}	0.2 – 6.2 x 10^{13}	-	137
**Coated wall (16), 245 K**	0.08 - 3.5 x 10^{11}	0.03 – 5.0 x 10^{13}	-	100
**Coated wall (3), 258 K**	0.4 – 0.5 x 10^{11}	0.2 – 0.3 x 10^{13}	-	46
**MC-DIFT, 253 K**	1.4 x 10^{12}	1.8 x 10^{14}	64	303
**MC-DIFT, 253 K**	1.7 x 10^{12}	2.0 x 10^{14}	95	199
**MC-DIFT, 253 K**	2.2 x 10^{12}	2.6 x 10^{14}	65	270
**MC-DIFT, 253 K**	2.2 x 10^{12}	1.5 x 10^{14}	73	245
**PC-DIFT, 253 K**	2.2 x 10^{12}	1.2 x 10^{14}	43	365
**PC-DIFT, 253 K**	2.2 x 10^{12}	1.5 x 10^{14}	58	349
**PC-DIFT, 253 K**	2.5 x 10^{12}	2.6 x 10^{14}	53	372
**PC-DIFT, 253 K**	2.8 x 10^{12}	1.7 x 10^{14}	58	315

References

(1) Abbatt, J. P. D. Interaction of HNO3 with water-ice surfaces at temperatures of the free troposphere. *Geophys. Res. Lett.* **1997**, *24* (12), 1479-1482. DOI: 10.1029/97GL01403.
(2) Hynes, R. G.; Cox, R. A. The interaction of HCl with water-ice at tropospheric temperatures. *Geophys. Res. Lett.* **2001**, *28* (14), 2827-2830.
(3) Huthwelker, T.; Ammann, M.; Peter, T. The Uptake of Acidic Gases on Ice. *Chem. Rev.* **2006**, *106* (4), 1375-1444. DOI: 10.1021/Cr020506v.
(4) Davidovits, P.; Kolb, C. E.; Williams, L. R.; Jayne, J. T.; Jayne, J. T.; Worsnop, D. R. Update 1 of: Mass Accommodation and Chemical Reactions at Gas−Liquid Interfaces. *Chem. Rev.* **2011**, *111* (4). DOI: 10.1021/cr100360b.
(5) Ammann, M.; Pöschl, U. Kinetic model framework for aerosol and cloud surface chemistry and gas-particle interactions - Part 2: Exemplary practical applications and numerical simulations. *Atmos. Chem. Phys.* **2007**, *7* (23), 6025-6045. DOI: 10.5194/acp-7-6025-2007.
(6) Pöschl, U.; Rudich, Y.; Ammann, M. Kinetic model framework for aerosol and cloud surface chemistry and gas-particle interactions - Part 1: General equations, parameters, and terminology. *Atmos. Chem. Phys.* **2007**, *7* (23), 5989-6023. DOI: 10.5194/acp-7-5989-2007.
(7) Hanson, D. R. Surface-Specific Reactions on Liquids. *J. Phys. Chem. B* **1997**, *101* (25), 4998-5001. DOI: 10.1021/jp970461f.
(8) Huthwelker, T.; Malmström, M. E.; Helleis, F.; Moortgat, G. K.; Peter, T. Kinetics of HCl Uptake on Ice at 190 and 203 K: Implications for the Microphysics of the Uptake Process. *J. Phys. Chem. A* **2004**, *108* (30), 6302-6318. DOI: 10.1021/jp0309623.
(9) Crowley, J. N.; Ammann, M.; Cox, R. A.; Hynes, R. G.; Jenkin, M. E.; Mellouki, A.; Rossi, M. J.; Troe, J.; Wallington, T. J. Evaluated kinetic and photochemical data for atmospheric chemistry: Volume V – heterogeneous reactions on solid substrates. *Atmos. Chem. Phys.* **2010**, *10* (18), 9059-9223. DOI: 10.5194/acp-10-9059-2010.
(10) Worsnop, D. R.; Zahniser, M. S.; Kolb, C. E.; Gardner, J. A.; Watson, L. R.; Vandoren, J. M.; Jayne, J. T.; Davidovits, P. Temperature-Dependence of Mass Accommodation of So2 and H2o2 on Aqueous Surfaces. *J. Phys. Chem.* **1989**, *93* (3), 1159-1172. DOI: DOI 10.1021/j100340a027.
(11) Pinzer, B. R.; Kerbrat, M.; Huthwelker, T.; Gäggeler, H. W.; Schneebeli, M.; Ammann, M. Diffusion of NOx and HONO in snow: A laboratory study. *J. Geophys. Res.* **2010**, *115* (D3), D03304. DOI: 10.1029/2009JD012459.
(12) Kerbrat, M.; Huthwelker, T.; Gäggeler, H. W.; Ammann, M. Interaction of Nitrous Acid with Polycrystalline Ice: Adsorption on the Surface and Diffusion into the Bulk. *J. Phys. Chem. C* **2010**, *114* (5), 2208-2219. DOI: 10.1021/jp909535c.
